# Evaluation of cerebrovascular reactivity using transcranial Doppler in patients with influenza

**DOI:** 10.1371/journal.pone.0340768

**Published:** 2026-01-12

**Authors:** Ana Orsolic Beslic, Kresimir Caljkusic, Antonela Matana, Katarina Tomelic Ercegovic, Mirela Pavicic Ivelja

**Affiliations:** 1 Department of Infectious Diseases, University Hospital of Split, Split, Croatia; 2 Department of Neurology, University Hospital of Split, Split, Croatia; 3 University of Split School of Medicine, Split, Croatia; 4 Faculty of of Health Sciences, University of Split, Split, Croatia; 5 Department for School and Adolescent Medicine, Public Health Institute of Split and Dalmatian County, Split, Croatia; James Cook University Hospital, UNITED KINGDOM OF GREAT BRITAIN AND NORTHERN IRELAND

## Abstract

Influenza is primarily a respiratory disease but can cause a broad spectrum of complications, including those affecting the cerebrovascular system. The aim of this study was to evaluate cerebrovascular reactivity in patients during and after influenza infection. A total of 92 participants, mean age 43 years, were enrolled; 46 patients with confirmed influenza infection and 46 healthy controls. Cerebrovascular reactivity was assessed using transcranial Doppler in combination with the breath-holding test and quantified by the breath-holding index (BHI). The influenza group demonstrated significantly lower cerebral blood flow velocities in the middle cerebral artery both at rest (PSVrest, MVrest, *p* < 0.001) and after the breath-holding test (PSVmax, MVmax, *p* < 0.001) compared to healthy controls. The BHI was also significantly reduced in the influenza group (*p* < 0.001), indicating impaired cerebrovascular reactivity. Among participants reassessed three months post-recovery, blood flow velocities after the breath-holding test were significantly higher than during the acute phase (PSVmax, MVmax, *p* < 0.001), and BHI also improved (*p* < 0.001), suggesting restoration of cerebrovascular function. These findings suggest that influenza may transiently impair cerebrovascular reactivity, providing new insights into potential mechanisms influencing cerebrovascular function and hemodynamics.

## Introduction

Influenza is an acute respiratory illness caused by influenza viruses, primarily types A and B, which are responsible for seasonal epidemics and, occasionally, global pandemics. Each year, influenza affects approximately 10% to 20% of the human population and is associated with up to 300,000 deaths worldwide, representing a major public health concern [1–4].

Influenza infection can lead to a variety of extra-pulmonary complications, including neurological manifestiations such as influenza‐associated encephalopathy, post-influenza encephalitis, Reye’s syndrome and Guillain-Barré syndrome, as well as myocarditis, rhabdomyolysis and cerebrovascular events, which represent an under-recognized burden of influenza. Early recognition of these manifestations, particularly cerebrovascular incidents, is essential for prevention and timely intervention [4].

Cerebrovascular disease is the second leading cause of death worldwide [5–7] and the leading cause of serious long**-**term disability in adults [8]**.** Therefore, it is essential to identify and manage stroke risk factors [5]. While conventional risk factors such as arterial hypertension, diabetes mellitus, obesity, hyperlipidemia, alcohol consumption and cigarette smoking are well established [6–10], an accumulating body of evidence indicates that infectious agents may also contribute to elevated cerebrovascular risk [5–7,11,12]. Multiple studies have shown that respiratory tract infections in general are associated with a transiently increased risk of vascular events, especially ischemic stroke and myocardial infarction [8,13–20]. In particular, influenza was found to be associated with a higher prevalence of stroke. Epidemiological studies have shown that the incidence of cardiovascular and cerebrovascular diseases, including stroke, as well as hospitalization and mortality rates due to these conditions rise significantly during influenza epidemics [5,12,20–23]. Moreover, numerous studies have demonstrated that influenza vaccination and use of antiviral drug oseltamivir are associated with a reduced incidence of cerebrovascular events suggesting a potential protective effect [24–26]. Although there is epidemiological and animal model evidence suggesting a potential link between influenza infection and the development of cerebrovascular diseases, strong conclusive evidence is still lacking. Pathophysiologically, it is hypothesized that influenza may contribute to cerebrovascular disease by causing endothelial injury and that the effect is likely mediated through systemic release of proinflammatory cytokines and other inflammmatory mediators leading to a prothrombotic state, inflammation-induced endothelial injury, or direct viral effects on the endothelium [8,23,27].

Cerebral blood flow autoregulation mechanisms are capable of maintaining stable cerebral blood supply despite fluctuations in cerebral perfusion allowing the brain to preserve adequate and constant perfusion necessary for maintaining metabolic homeostasis [7,28,29]. Transcranial Doppler (TCD) ultrasonography is a real-time, noninvasive and well-tolerated method for evaluating hemodynamic parameters and flow rates of main basal intracranial arteries**.** It enables the measurement of blood flow velocities (BFV) and cerebrovascular resistances [7,30,31,33]. Cerebral vasoreactivity represents the capacity of cerebral blood vessels to respond to vasodilatory stimuli such as breath-holding, carbon dioxide (CO₂) inhalation, or acetazolamide administration by altering blood flow velocity. Among these, the breath-holding test (BHT) in combination with TCD represents a real-time, noninvasive and and well-tolerated method for evaluating cerebrovascular hemodynamics and reactivity, The middle cerebral artery (MCA), which carries the majority of cerebral blood flow, is particularly suitable for measurement by TCD via the temporal bone window [7]. The Breath-Holding Index (BHI) serves as a quantitative measure of cerebrovascular reactivity. Assessment of vasoreactivity provides valuable information on the cerebrovascular reserve capacity, which reflects the ability of cerebral vessels to adjust to systemic hemodynamic changes or increased metabolic demand. Impaired cerebrovascular reactivity, characterized by a reduced capacity to increase cerebral blood flow in response to vasodilatory stimuli, has been observed in several clinical conditions associated with cerebrovascular disease [7,30–33]. Although previous studies have demonstrated an association between influenza infection and an increased prevalence of stroke, definitive evidence supporting this relationship is still lacking. The aim of this study was to evaluate cerebrovascular reactivity in patients during and after influenza infection using TCD in combination with the breath-holding test.

We hypothesized that cerebral vasoreactivity is transiently impaired during the acute phase of influenza infection.

## Materials and methods

### Study design, participants and data collection

We conducted a prospective cohort study. The study was approved by the Ethics Committee of the University Hospital Split in August 2023 (class 500–03/23–01/170, NO 2181–147/01/06/LJ.Z-23–03.). Before the collection of any data, all patients signed an individual written informed consent form. The study included 92 Caucasian adults, aged 28–62 years, divided into two groups. The influenza group consisted of 46 participants who presented to the Department of Infectious Diseases, University Hospital of Split with respiratory symptoms and a positive rapid antigen test for influenza. All acute-phase measurements were performed on the day of presentation. Only patients presenting within the first four days of symptom onset were included, ensuring that evaluations occurred during the acute phase of illness. The control group included 46 healthy volunteers who had no symptoms of infectious disease and who were negative for influenza rapid antigen test at the time of enrollment. Our study relied on clinical presentation combined with rapid antigen testing, following methodologies used in previous clinical and outbreak settings, such as Spanish H1N1 cohort, institutional outbreaks in Ontario, and a meta–analysis of H1N1 studies, where rapid antigen test demonstrated high specificity (100%, 99.7%, and 98%) and positive predictive value in symptomatic individuals, supporting their use for diagnostic classification [34–36]. All participants were tested with the Rapid Influenza Antigen Nasal Test Kit (Ecotest, Hague, The Netherlands) during the period from January 1. to June 30. 2024, which corresponds to the Northern Hemisphere influenza season [37]. Each individual underwent a medical history assessment, physical examination, blood pressure measurement, and TCD ultrasonography with breath-holding test. Approximately three months after initial assessment, the influenza group underwent a repeat TCD examination using the same protocol.

Participant data included: age, sex, height, weight, body mass index (BMI), past and current diseases, regular medication use, smoking and alcohol use, amount of physical activity, comorbidities (including diabetes mellitus, arterial hypertension, atrial fibrillation, active malignant disease, coronary heart disease, liver cirrhosis, prior cerebrovascular disease, hyperlipidemia, and depression). They were asked for history of influenza vaccination and past influenza infections. For each participant the Charlson Comorbidity Index (CCI) was calculated. The amount of physical activity was assessed using the International Physical Activity Questionnaire and are expressed as a minutes of moderate physical activity per week. Exclusion criteria included participants younger than 28 years, due to the potentially increased elasticity of cerebral blood vessels that may result in higher flow velocities, and those older than 65 years, due to increased vascular resistance and arterial stiffness. Other exclusion criteria were risk factors for cerebrovascular disease including: obesity (BMI > 30), arterial hypertension, diabetes mellitus, active malignant disease or hematologic disease, atrial fibrillation or chronic heart disease, cerebrovascular disease in anamnesis, liver chyrosis, heavy alcohol consumption (more than 7 drinks per week for women and 14 for men), drug use, known occlusive disease of cerebral arteries, stenosis of the vertebral artery or external carotid artery more than 20% and CCI > 2. We excluded all patients using α-blockers, β-blockers, and calcium channel blockers, vasodilators, anticoagulant or hormone replacement therapy. Control participants were manually selected to match each influenza participant based on predefined criteria within the following tolerance ranges: age ± 3 years, BMI ± 2 kg/m^2^, systolic and diastolic blood pressure ±10 mmHg, same sex, comparable physical activity level (low/moderate/high), and lifestyle habits (smoking status and alcohol consumption). This approach ensured comparable baseline characteristics between groups. Propensity score matching was not used. None of the participants in the influenza group were hospitalized or treated as outpatients for influenza pneumonia. Due to inadequate insonation of the MCA, one participant from the influenza group was excluded. The sample size was determined based on the number of eligible patients presenting during the study period and is comparable to previous studies evaluating cerebrovascular reactivity using TCD [6,7]. As this investigation was designed as an observational exploratory study, a formal power calculation was not performed.

The primary outcome of the study was cerebrovascular reactivity, expressed as the BHI, compared between the influenza and control groups. The secondary outcome was comparison of BHI within the influenza group between the acute phase and three months post-recovery.

**Table:** 

Exclusion Critera
Age under 28 and over 65 years
Obesity (BMI > 30)
Severe or critical form of influenza infection
History of: arterial hypertension, diabetes mellitus, active malignant disease or hematologic disease, atrial fibrillation or chronic heart disease, cerebrovascular disease in anamnesis, liver chyrosis
Heavy alcohol consumption (>7 drinks for women and >14 for men per week) or substance abuse
Known occlusive disease of cerebral arteries
Stenosis of the vertebral artery or external carotid artery more than 20%
Using: α-blockers, β-blockers, calcium channel blockers, vasodilator, anticoagulant or hormone replacement therapy
Charlson Comorbidity Index>2

### Procedure and measurements

We performed all measurements using the Transcranial Doppler Multi-Dop T Ultrasound system (Compumedics DWL, Germany). All participants were examined during the afternoon in a quiet room while lying in a supine position. Initial measurements were performed in the resting phase, which lasted 5 minutes during which we recorded baseline cerebral blood flow velocities. Following this, participants were asked to take a normal inspiration and then hold their breath for as long as they could. After the breath-holding period, they breathed normally for 5 minutes. We repeated the procedure for each participant three times. We insonated the right MCA using a 2.0 MHz pulsed Doppler probe at a depth of 48–62 mm via right temporal bone window. For each participant, we measured blood flow velocities in the right middle cerebral artery, including peak systolic velocity (PSV) and mean velocity (MV), as well as cerebrovascular resistance indices: Gosling’s Pulsatility Index (PI) and the Resistance Index (RI), also known as the Pourcelot Index [7,30,31,33]. Measurements were obtained at rest and recorded as PSV rest, MV rest, RI rest and PI rest, and three times after the breath–holding test, recorded as PSV max, MV max, RI max and PI max. We determined the mean values of each variable from the three measurements taken after the breath-holding test. We also measured the time of breath-holding (TBH). Cerebrovascular reactivity was assessed by calculating the BHI**,** defined as the percent increase in mean blood flow velocity from baseline during breath holding, divided by the duration of breath holding in seconds. All TCD examinations, including those performed during the acute-phase and three months post-recovery, were performed by the same experienced sonographer to minimize inter-operator variability. The procedure used in our study has previously been applied in multiple studies, including those by Pavicic Ivelja et al., Marcic et al., and Settakis et al. [6,7,38].

### Statistical analysis

The distribution of continuous variables was examined using the one-sample Kolmogorov–Smirnov test. Variables following a normal distribution are presented as mean ± standard deviation (SD), whereas those with a non-normal distribution are expressed as median and interquartile range (IQR). Categorical variables are described using frequencies and percentages. Group differences were assessed, as appropriate, with the chi-square test for categorical variables and with either Student’s t-test or the Mann–Whitney U test for continuous variables. Comparisons of paired measurements were performed using the paired t-test for normally distributed data and the Wilcoxon signed-rank test for data that did not meet the assumption of normality. Associations between two continuous variables were evaluated using Pearson’s correlation analysis, while associations between continuous and dichotomous variables were assessed using the point–biserial correlation coefficient. All statistical analyses were performed using JASP (Version 0.18.3). A p-value &lt; 0.05 was considered statistically significant.

## Results

### Participants characteristics

This study included 92 participants equally divided into two groups. Both groups were matched by age (independent t-test, df = 90, *p* = 0.954), sex (chi-square test, df = 1, *p* = 1.000), BMI (Mann-Whitney test, df = 49, *p* = 0.441), smoking status (chi-square test, df = 1, *p* = 1.000) and alcohol consumption habits (not applicable). The mean age in both groups was 43 years, including 19 (41%) male and 27 (59%) female participants in each group. All participants had Charlson Comorbidity Index score below 2.

A statistically significant difference was observed between groups regarding weekly physical activity levels over the seven days preceding the assessment. The influenza group had a significantly lower level of physical activity, expressed in minutes per week, compared to the control group (Mann-Whitney test, df = 90, *p* < 0.001).

There were no statistically significant differences between the groups regarding influenza vaccination during the current season (chi-square test, df = 1, *p* = 0.557) or history of previous influenza infections (chi-square test, df = 1, *p* = 0.788). Baseline characteristics are presented in Table 1.

**Table 1:** Baseline characteristics of influenza and control group participants.

	Influenza group (n = 46)	Control group (n = 46)	*p*-value
Age, years (mean, ± SD)	43 ± 8.9	43 ± 9.1	0.954*
Male sex (n, %)	19 (41)	19 (41)	1.000 **
BMI, kg/m^2^ (median, IQR)	26 (22.7–27.6)	25 (22.8–27.8)	0.441^†^
Smokers (n, %)	16 (34)	16 (34)	1.000**
Alcohol consumption <7 beverages per week (n, %)	46 (100)	46 (100)	NA ‡
BP systolic, mmHg (median, IQR)	120 (120–120)	120 (120–120)	0.351^†^
BP diastolic, mmHg (median, IQR)	80 (80–80)	80 (80–80)	0.837^†^
CCI score<2 (n,%)	46 (100)	46 (100)	NA ‡
Physical activity (minutes per week of moderate activity;median, IQR)	100 (100–120)	180 (160–180)	<0.001^†^
This year influenza vaccination (n,%)	1 (2)	2 (4)	0.557**
Previous influenza infection (n,%)	8 (17)	9 (19)	0.788**

*independent t-test; **chi-square test; † Mann-Whitney test; ‡NA = not applicable; SD = standard deviation; IQR = interquartile range; BMI = body mass index; BP = blood pressure; CCI = Charlson Comorbidity Index.

### Disease symptoms in influenza group

The most common symptoms reported in the influenza group (n = 46) were a non-productive cough reported by 45 participants (97%), followed by chills in 44 (95%), general malaise in 41 (89%), fever in 34 (74%), headache in 34 (74%), and rhinorrhea in 36 (78%). None of the participants were hospitalized due to influenza infection (Table 2).

**Table 2:** Most common influenza–related symptoms in influenza group.

Symptom	Influenza group (n = 46), n (%)
Fever (> 38.5 °C)	34 (74)
Chills	44 (95)
Shivering	19 (41)
General malaise	41 (89)
Headache	34 (74)
Rhinorrhea	36 (78)
Sore throat	25 (54)
Nausea	18 (39)
Vomiting	5 (10)
Cough	45 (97)
Shortness of breath	13 (28)
Chest pain	6 (13)

°C = degrees Celsius.

### Comparison of cerebrovascular parameters and BHI between influenza and control groups

There were no clinically or statistically significant differences (*p* > 0.05) between the three repeated measurements after the breath-holding test in either group, so we calculated the arithmetic mean of these measurements for each participant and used it in further analyses.

At the rest, influenza group had statistically significantly lower PSVrest (independent t-test, df = 89, *p* < 0.001) and MVrest (independent t-test, df = 89, *p* < 0.001) compared to the control group. There was no statistically significant difference in PIrest (Mann-Whitney test, df = 87, *p* = 0.551) or RIrest (Mann-Whitney test, df = 89, *p* = 0.768) between the groups. After the breath-holding test, both groups had higher velocity parameters and lower resistance indices compared to the rest period. Influenza group had statistically significantly lower PSVmax (Mann-Whitney test, df = 89, *p* < 0.001), MVmax (Mann-Whitney test, df = 89, *p* < 0,001), and higher PImax (Mann-Whitney test, df = 89, *p* = 0.033) compared to control group. We did not find statistically significant difference for RImax (Mann-Whitney test, df = 89, *p* = 0.128). The median breath-holding time in the influenza group was 35 seconds (IQR 32–40), which was significantly shorter than in the control group, where median was 39.5 seconds (IQR 36–45; Mann-Whitney test, df = 89, *p* = 0.004). Relative increases in flow velocities after the breath**-**holding test were statistically significantly higher in the control group compared to the influenza group for both PSV and MV. Specifically, PSV was 3.23 times higher in the control group, with a median difference of 24.5 (Mann–Whitney test, df = 90, *p* < 0.001), and MV was 3.5 times higher, with a median difference of 25 (Mann-Whitney test, df = 90, *p* < 0.001). A statistically significant difference was also observed in the change in pulsatility index (∆PI), which was lower in the control group compared to the influenza group (median difference: 0.01; Mann-Whitney test, df = 90, *p* = 0.014). However, no significant difference was found in the change in resistance index (∆RI) between the groups (Mann-Whitney test, df = 90, *p* = 0.76). Vascular reactivity, as measured by the BHI was significantly higher in the control group compared to the influenza group, with a median difference of 0.52 (Mann-Whitney test, df = 90, *p* < 0.001). Data are presented as mean ± SD and as median (IQR) in Table 3.

**Table 3:** Cerebral blood flow velocities at rest and after the breath-holding test, and BHI, in the influenza and control groups.

	Influenza group (n = 46)	Control group (n = 46)	*p*-value
**Participants at rest**			
PSV (cm/s)	102 ± 22.8	122 ± 13.5	<0.001*
MV (cm/s)	70.2 ± 18.04	83.76 ± 10.08	<0.001*
PI	0.78 (0.69–0.85)	0.80 (0.72–0.85)	0.551^†^
RI	0.53 (0.50–0.58)	0.54 (0.52–0.56)	0.768^†^
**Participants after breath-holding test**			
PSV (cm/s)	110 (98–135)	158 (147–173)	<0.001^†^
MV (cm/s)	79 (69–98)	115 (109–129)	<0.001^†^
PI	0.67 (0.63–0.77)	0.64 (0.59–0.69)	0.033^†^
RI	0.49 (0.46–0.53)	0.48 (0.45–0.50)	0.128^†^
TBH (s)	35 (32–40)	39.5 (36–45)	0.004 ^†^
**Cerebrovascular reactivity parameters**			
∆PSV (%)	11 (9–16.5)	35.5 (27.5–39.75)	<0.001^†^
∆MV (%)	10 (6–14)	35 (30.25–40)	<0.001^†^
∆PI (%)	−0.11 (**−**0.18– − 0.06)	−0.12 (**−**0.15– − 0.06)	0.014^†^
∆RI (%)	−0.06 (**−**0.09– − 0.03)	−0.06 (−0.09– − 0.04)	0.76^†^
BHI	0.47 (0.41–0.54)	0.99 (0.91–1.04)	<0.001^†^

*Independent t-test; † Mann-Whitney test; PSV = peak systolic velocity; MV = mean velocity; PI = pulsatility index; RI = resistance index; TBH = time of breath holding; BHI = breath holding index.

### Correlation of cerebral flow parameters with age for influenza and control group

Pearson’s correlation coefficient (r) was used to assess the relationship between age and cerebral hemodynamic parameters separately in the influenza and control groups.

In the influenza group, significant negative correlations with age were observed for all measured flow velocities, both at rest and after the breath-holding test, including: PSVrest (r = –0.56, *p* < 0.001), MVrest (r = –0.49, *p* = 0.001), PSVmax (r = –0.53, *p* < 0.001), MVmax (r = –0.53, *p* < 0.001). No significant correlations were observed for PIrest, RIrest, PImax or Rimax. In the control group**,** no statistically significant correlations were found between age and any of the measured hemodynamic parameters.

**Table 4:** Correlation between age and cerebral hemodynamic parameters in influenza and control group.

	Influenza group (n = 46)		Control group (n = 46)	
	r	*p*-value	r	*p-*value
PSVrest (cm/s)	−0.56	<0.001	0.26	0.076
MVrest (cm/s)	−0.49	0.001	0.23	0.130
PIrest	0.08	0.619	0.10	0.530
RIrest	0.13	0.379	0.18	0.219
PSVmax (cm/s)	−0.53	<0.001	0.10	0.498
MVmax (cm/s)	−0.53	<0.001	0.09	0.546
Pimax	0.29	0.058	0.06	0.670
Rimax	0.22	0.144	0.07	0.626

r = Pearson’s correlation coefficient; PSV = peak systolic velocity; MV = mean velocity; PI = pulsatility index; RI = resistance index.

### Correlation of cerebral flow parameters with sex for influenza and control group

The association between sex and cerebral hemodynamic parameters in influenza group was assessed using the point-biserial correlation coefficient (*r*).

No significant correlations were found between sex and any of the hemodynamic parameters in either group (all *p* > 0.05).

**Table 5:** Correlation between gender and hemodynamic parameters in influenza and control groups.

	Influenza group (n = 46)		Control group (n = 46)	
	r	*p*-value	r	*p*-value
PSVrest (cm/s)	0.15	0.333	−0.004	0.977
MVrest (cm/s)	0.21	0.171	0.20	0.179
PIrest	−0.24	0.108	−0.21	0.157
RIrest	−0.14	0.367	−0.02	0.883
PSVmax (cm/s)	0.17	0.269	0.18	0.229
MVmax (cm/s)	0.21	0.172	0.21	0.155
PImax	−0.04	0.795	0.01	0.954
RImax	0.08	0.619	0.09	0.563

PSV = peak systolic velocity; MV = mean velocity; PI = pulsatility index; RI = resistance index.

### Comparison of cerebrovascular parameters and BHI in influenza participants during the acute phase and three months post-recovery

At rest, there were no statistically significant differences in any of the measured velocity parameters within the influenza group between the acute phase and three months post-recovery. After the breath-holding test, there were significantly lower PSVmax (Wilcoxon signed-rank test, df = 87, *p* < 0.001) and MVmax (Wilcoxon signed-rank test, df = 87, *p* < 0.001) in acute phase compared to the post-recovery period. However, no significant differences were observed in the PImax (Wilcoxon signed-rank test, df = 86, *p* = 0.066), and RImax (Wilcoxon signed-rank test, df = 87, *p* = 0.216) between the two time points. The mean breath-holding time was significantly shorter during the acute phase, 35 seconds (IQR 32–40), compared to the three month post-recovery period, when it increased to 39 seconds (IQR 35–44) (Wilcoxon signed-rank test, df = 86, *p* < 0.001). There was a significant increase in weekly physical activity in the post–recovery period compared to the acute phase, with values of 181.6 ± 6.1 minutes and 108.3 ± 13 minutes (Wilcoxon signed-rank test, df = 88, *p* < 0.001). Relative increases in flow velocities after the breath-holding test were statistically significantly higher in the post-recovery period compared to the acute phase of influenza with PSV 2.5 times higher and median difference of 17 (Wilcoxon signed-rank test, df = 90, *p* < 0.001), and MV 2.3 times higher, with a median difference of 13.5 (Wilcoxon signed-rank test, df = 90, *p* < 0.001). No significant differences were observed in the change of pulsatility index (∆PI; Wilcoxon signed-rank test, df = 90, *p* = 0.060) or resistance index (∆RI; Wilcoxon signed-rank test, df = 90, *p* = 0.866) between two time points. The BHI was significantly higher in the post recovery period with a median difference of 0.44 (Wilcoxon signed-rank test, df = 87, *p* < 0.001). Data are presented as mean ± SD and median (IQR) in Table 6.

**Table 6:** Cerebral blood flow velocities at rest and after the breath-holding test, and BHI, in influenza group during the acute phase and three months post–recovery.

Influenza group, (n = 46)	Acute phase	Post-recovery	*p*-value
**Participants at rest**			
PSV (cm/s)	102 ± 22.8	103 ± 20.6	0.861*
MV (cm/s)	66 (56–85)	70.5 (59–81)	0.615^†^
PI	0.78 (0.69–0.85)	0.78 (0.71–0.86)	0.834^†^
RI	0.53 (0.50–0.58)	0.54 (0.51–0.57)	0.741^†^
**Participants after breath-holding test**			
PSV (cm/s)	110 (98–135)	138 (115–149)	<0.001^†^
MV (cm/s)	79 (69–98)	96.5 (81–113)	<0.001^†^
PI	0.67 (0.63–0.77)	0.63 (0.60–0.71)	0.066^†^
RI	0.49 (0.46–0.53)	0.48 (0.45–0.51)	0.216^†^
TBH (s)	35 (32–40)	39 (35–44)	<0.001^†^
**Cerebrovascular reactivity parameters**			
∆PSV (%)	11 (9–16)	28 (22–33)	<0.001^†^
∆MV (%)	10.5 (8–17)	24 (22–31)	<0.001^†^
∆PI (%)	−0.09 (−0.17– − 0.04)	−0.11 (−0.20– − 0.06)	0.060^†^
∆RI (%)	−0.06 (−0.10– − 0.03)	−0.04 (−0.09– − 0.02)	0.866^†^
BHI	0.47 (0.41–0.54)	0.91 (0.85–1.00)	<0.001^†^

*Paired t-test; † Wilcoxon signed-rank test; PSV = peak systolic velocity; MV = mean velocity; PI = pulsatility index; RI = resistance index; TBH = time of breath holding; BHI = breath holding index.

## Discussion

Our study demonstrated that even mild influenza infection may transiently impair cerebrovascular reactivity in individuals without conventional risk factors for cerebrovascular disease, suggesting a potential impact of influenza on cerebrovascular function.

The basic principle of cerebral vasoreactivity is the dilation of small blood vessels, which leads to increased flow velocities in the large basal cerebral arteries [39]. This response occurs during hemodynamic changes or increased metabolic demand. After the breath**-**holding test, flow velocities in these arteries increase due to induced hypercapnia and hypoxia [38]. Our study observed that flow velocities through the middle cerebral artery, including peak systolic velocity (PSV) and mean velocity (MV), were significantly lower in influenza group both at rest and after the breath-holding test compared to the control group. Consequently, relative changes in flow velocities (∆PSV and ∆MV) were also significantly reduced in the influenza group (Table 3), indicating reduced cerebral perfusion. The pulsatility index, which reflects resistance in distal cerebral vasculature [40–41], was significantly higher in influenza group after the breath-holding test compared to the control group. Additionally, the relative change in PI was also significantly higher in influenza group (Table 3), suggesting that influenza may be associated with increased cerebrovascular resistance. Finally, the influenza group had significantly lower BHI values compared to the control group (Table 3), reflecting impaired cerebrovascular reactivity, which has been recognized as a potential precursor to cerebrovascular disease [6,29,30]**.** To date, no studies have directly investigated cerebrovascular reactivity in patients with influenza. Our results are consistent with previous epidemiological research demonstrating that influenza increases the risk of cerebrovascular events [12,20,22,23]. For example, Toschke *et al*. reported a significant rise in ischemic stroke cases within two weeks and in hemorrhagic strokes within four weeks after the seasonal influenza peak [21]. Furthermore, numerous studies [5,13,24–26,42–44], including a population-based study from an entire Canadian province by Holodinsky *et al.* [17] and a meta-analysis by Lee *et al.* [45] have demonstrated a significant association between recent influenza vaccination and a reduction in incidence of all types of stroke. A meta-analysis by Tavabe *et al.* showed that influenza vaccination reduces the risk of stroke and stroke-related hospitalization by approximately 16% [5]. Similarly, a population-based case-control study by Lin *et al.* demonstrated that receiving the influenza vaccine during the current season was associated with a 24% reduction in the risk of ischemic stroke. Moreover, consistent annual vaccination over a five-year period was linked to a reduced risk of hospitalization due to ischemic stroke [26].

We also compared measurements of blood flow velocities during acute influenza infection and approximately three months post-infection, in the same individuals. Our findings demonstrated significantly higher blood flow velocities after the breath-holding test in the post-recovery period, including both PSV and MV, accompanied by greater relative changes in flow velocities (∆PSV and ∆MV; Table 6). Importantly, BHI also improved after recovery, reflecting restored cerebrovascular reactivity (Table 6). In contrast, resting cerebral blood flow velocities did not normalize within the three-month follow-up period, suggesting that restoration of basal cerebral perfusion may require a longer recovery period than cerebrovascular reactivity. Because our study included only a three-month follow-up, it remains uncertain whether full normalization could occur at later time points. Longer-term studies are needed to determine whether basal perfusion ultimately returns to baseline and to identify the timeframe over which perfusion recovery occurs. We observed that the BHT was significantly shorter in influenza group compared to controls, likely due to respiratory limitations associated with the infection. Despite these differences, all participants achieved BHT values sufficient to induce hypercapnia, as reflected by measurable increases in cerebral blood flow velocities. Consistently, Settakis et al. reported that cerebral blood flow velocity increases after 30 seconds of breath-holding, and Markus and Harrison observed a mean breath-holding duration of 31.5 seconds sufficient to induce measurable cerebrovascular responses [30,38]. In our study, the influenza group achieved a mean BHT of 35 seconds (range 32–40 s), exceeding the durations reported in these studies, Improvement of both BHT and BHI at three months post-recovery indicates that the initially reduced BHI was largely due to transient cerebrovascular impairment. This highlights that influenza temporarily affects cerebrovascular reactivity, which recovers following resolution of the infection.

Our observations aligns with several studies [13,21,22,46], including that by Boehme *et al.,* who reported that the highest odds of stroke occur within two weeks following an influenza-like illness (ILI), with the risk gradually declining and returning to baseline approximately two months later [8]. Furthermore, a study by Kulick *et al.* demonstrated a 39% increase in the odds of stroke and a 24% increase in the odds of myocardial infarction (MI) during the first 15 days after ILI [18]. These findings suggest that influenza may act as a short**-**term trigger for cerebrovascular events, which is consistent with our results.

Conventional stroke risk factors are estimated to account for only 50–80% of stroke incidence [12,22], and cerebrovascular events also occur in individuals without any of these risk factors, particularly in younger and middle-aged populations [6,10]. Our study included middle-aged participants (mean age 43 years) with Charlson Comorbidity Index scores below 2, ensuring a relatively healthy study population. We excluded individuals with conventional stroke risk factors to eliminate other potential factors that can contribute to cerebrovascular disease. This allowed for a more accurate assessment of the impact of influenza on cerebral vasoreactivity, revealing that patients in this population showed significant changes in vascular responsiveness. These findings align with those of Boehme *et al.,* who reported that younger patients, under the age of 45, had a relatively increased risk of stroke after ILI and that for every decade decrease in age, the odds increased by almost 10% [8].

Previous studies have shown that aging is independently associated with reductions in cerebral perfusion [47–49]. Tomoto *et al.* demonstrated that cerebral blood flow decreases by approximately 3.5 mL/min per year [50]. In our study, we found that cerebral flow velocities declined with age in influenza group, while they remained stable in healthy controls (Table 4). The absence of significant correlations in the control group indicates that cerebral hemodynamics are relatively preserved with age in healthy middle-aged individuals. In contrast, moderate, but statistically significant age-related declines observed in influenza group were likely clinically detectable due to the additive cerebrovascular impact of influenza infection.

Sex-related differences in cerebral hemodynamics have also been well documented. Women have higher cerebral flow velocities compared to men [47,51]. Gur *et al.* also reported that this sex-related difference diminishes by the sixth decade of life, after which men and women have similar flow rates. Moreover, the study by Kulick *et al.* found that the association between ILI and stroke was stronger in men and individuals living in rural areas, with no significant differences observed across racial or ethnic groups [18]. Vollmer *et al.* demonstrated that the association between stroke and ILI is strongest among younger individuals, particulary males, who had not received seasonal influenza vaccination [46]. In our study, male participants exhibited lower cerebral flow velocities compared to females within the influenza group. However, these differences were not statistically significant (Table 5).

Other infectious agents have similarly been associated with cerebrovascular endothelial dysfunction, including *Chlamydia pneumoniae*, *Helicobacter pylori,* cytomegalovirus, and periodontal pathogens, etc. [5–7,11,12]. These pathogens are thought to contribute to the development of cerebrovascular disease by promoting inflammation, causing changes in brain blood vessels through different mechanisms [10]. Pavicic Ivelja *et al.* demonstrated that patients with chronic hepatitis C have impaired cerebral vasoreactivity, which may negatively affect cerebrovascular hemodynamics and contribute to an increased risk of cerebrovascular disease [6]. Furthermore, Chow *et al*. found that HIV-infected individuals also had impaired cerebrovascular reactivity, suggesting increased cerebrovascular risk [52].

Influenza shares many clinical and pathophysiological features with SARS-CoV-2, including effects on inflammatory and coagulation pathways [1]. A study by Marcic *et al.* demonstrated impaired cerebrovascular reactivity in patients following COVID**-**19 infection. Repeat testing 300 days post**-**infection still revealed reduced cerebral vasoreactivity. Comparing to our findings, this suggests that SARS**-**CoV**-**2 may cause a more prolonged impairment of cerebrovascular reactivity than influenza infection and may have longer**-**lasting effects on cerebrovascular function [7,53].

The exact pathophysiological mechanisms by which influenza contributes to the development of cerebrovascular diseases remain unclear. However, several animal studies have demonstrated that influenza infection can lead to endothelial dysfunction, a key factor in cerebrovascular pathogenesis. Haidari *et al.* showed that the influenza virus can directly infect atherosclerotic arteries, promoting systemic and local inflammation [54]. Muhammad *et al.* found that influenza triggers a cytokine cascade that aggravates ischemic brain injury, independent of direct brain infection, fever, or hypoxemia, suggesting a cytokine mediated mechanism in influenza related stroke risk [27].

The main limitation of this study is that it was conducted at a single center and included only Caucasian participants with a relatively small sample size. Another limitation is that the quality of measurements obtained via TCD largely depends on the adequacy of the temporal bone window through which the middle cerebral artery is insonated. Additionally, the hemodynamic effect of breath-holding test is weaker than that produced by acetazolamide injection or carbon dioxide inhalation. We mitigated these limitations by excluding participants with conventional cerebrovascular risk factors and by carefully matching the influenza and control groups for age, gender, BMI, blood pressure, and lifestyle factors such as smoking and alcohol use.

Future studies should include larger patient cohorts and compare influenza patients who received antiviral therapy or influenza vaccination with those who did not, in order to evaluate the potential protective effects of these interventions against the risk of cerebrovascular incidents, and to validate findings in the context of current epidemiological evidence.

## Conclusions

Our findings contribute to the understanding of the relationship between influenza infection and cerebrovascular function by showing that influenza may transiently reduce cerebrovascular reactivity, providing insight into potential mechanisms that may influence cerebral hemodynamics. Notably, our study demonstrates that even mild cases of influenza can negatively affect cerebrovascular reactivity in individuals without conventional risk factors. These results highlight that influenza should be considered not only as a respiratory illness but also as a condition with potential cerebrovascular implications. Further research is needed to better understand the underlying mechanisms and long-term consequences of influenza on cerebrovascular health.
